# Effects of the Entomopathogenic Fungus *Metarhizium anisopliae* on the Mortality and Immune Response of *Locusta migratoria*

**DOI:** 10.3390/insects11010036

**Published:** 2019-12-31

**Authors:** Wuji Jiang, Yifan Peng, Jiayi Ye, Yiyi Wen, Gexin Liu, Jiaqin Xie

**Affiliations:** 1 Genetic Engineering Research Center, School of Life Sciences, Chongqing University, Chongqing 401331, China; 2 Chongqing Engineering Research Center for Fungal Insecticides, Key Laboratory of Gene Function and Regulation Technology under Chongqing Municipal Education Commission, Chongqing 401331, China

**Keywords:** *Metarhizium anisopliae*, mortality, *Locusta migratoria*, immune response, pest control

## Abstract

Entomopathogenic fungi are the key regulators of insect populations and some of them are important biological agents used in integrated pest management strategies. Compared with their ability to become resistant to insecticides, insect pests do not easily become resistant to the infection by entomopathogenic fungi. In this study, we evaluated the mortality and immune response of the serious crop pest *Locusta migratoria manilensis* after exposure to a new entomopathogenic fungus strain, *Metarhizium anisopliae* CQMa421. *M. anisopliae* CQMa421 could effectively infect and kill the *L. migratoria* adults and nymphs. The locust LT_50_ under 1 × 10^8^ conidia/mL concentration of *M. anisopliae* was much lower than that under conidial concentration 1 × 10^5^ conidia/mL (i.e., 6.0 vs. 11.2 and 5.0 vs. 13.8 for adults and nymphs, respectively). The LC_50_ (log_10_) of *M. anisopliae* against locust adults and nymphs after 10 days was 5.2 and 5.6, respectively. Although the number of hemocytes in *L. migratoria* after exposure to *M. anisopliae* did not differ with that in the controls, the enzymatic activity of superoxide dismutase (SOD) and prophenoloxidase (ProPO) did differ between the two treatments. The activities of both SOD and ProPO under the *M. anisopliae* treatment were lower than that in the controls, except for the ProPO activity at 72 h and the SOD activity at 96 h. Further, the expression of the *L. migratoria* immune-related genes defensin, spaetzle, and attacin differed after exposure to *M. anisopliae* for 24 h to 96 h. Taken together, this study indicated that infection with *M. anisopliae* CQMa421 could cause the death of *L. migratoria* by interacting with the immune responses of the host, demonstrating that this fungal strain of *M. anisopliae* can be an efficient biocontrol agent against *L. migratoria*.

## 1. Introduction

Entomopathogenic fungi are typically present within natural insect populations and are often solely considered as effective microbial control agents in integrated pest management [1,2,3,4]. The use of fungal insect pathogens may have certain advantages over the use of parasitoids and insecticides such as efficiency and environmental safety [5]. Several fungal agents have been used to control pests, such as the rice planthopper *Nilaparvata lugens*, *Haemaphysalis longicornis*, and the oriental migratory locust *Locusta migratoria manilensis* Meyen, and have achieved good results [6,7,8,9]. Currently, chemical insecticides are commonly used for insect pest control [10]. However, the misuse of such insecticides has caused destructive damage to the environment and human health [11,12]. The side effects on nontargets and the resurgence of insect pests have received much attention, and consequently, there is a growing trend to reduce the use of these chemical insecticides [13]. Moreover, the enhanced resistance of insect pests to many chemical insecticides has resulted in long-standing and expanding problems for pest arthropod control [14]. Compared with their ability to become resistant to insecticides, insect pest hosts do not easily become resistant to fungal infection, and entomopathogenic fungi have been used to control a few insecticide-resistant insect species [15].

Entomopathogenic fungi can infect insects, namely, the fungal conidia attach and penetrate through the insect’s cuticle, causing death. Such processes involve several physiological or immune responses of hosts to these types of xenobiotics and pathogens [16]. Although all invertebrates lack an adaptive immune response, they may defend against pathogens by relying on their innate immunity (i.e., cellular and humoral immune responses) [17,18,19]. In hosts, these xenobiotics can be countered by phagocytosis, or by the activation of the host innate immunity. However, entomopathogenic fungi can mask their cell wells to evade the immune system of insect hosts [20] and release chitinase, chitosanase, and lipase to suppress the host regulatory system [21]. In addition, these fungi can produce a few compounds such as beauvericin compounds and destruxins to paralyze the hosts [22]. Thus, the immune responses of hosts are important processes activated in response to the functions of xenobiotics or pathogens.

In response to challenge with insecticides or pathogens, many metabolic processes or immune responses within insect hosts are activated. The antioxidant enzyme of superoxide dismutase (SOD) is a key modulator of host immunity function and is associated with the phagocytotic ability and melanization of insects [23]. The activity of enzymes such as SOD can be stimulated by different insecticides and is associated with the resistance to chemical insecticides [24]. Prophenoloxidase (ProPO) is a crucial factor in the defense against pathogen or insecticide challenge [25]. Increased ProPO activity can enhance the insect immune system ability in response to xenobiotic challenges and can promote healing [19,26].

In *Drosophila melanogaster*, the Toll and IMD signaling pathways regulate the synthesis of immune effectors [27,28,29]. Unlike its mammalian counterparts, insect Toll is activated through an endogenous ligand and nerve growth factor-related cytokine spaetzle (a gene encoding a Toll-activating protease), but not by direct interaction with microbial molecules [30]. Insect defensins are cationic, cysteine-rice peptides (ca. 4 kDa), inducible antibacterial peptides that may appear after pathogenic challenge or injury in the insect hemolymph [31]. Attacin is an important antimicrobial peptide that is related to the humoral immune system of insect hosts [32]. The expression level of these defensin genes may reflect the immune responses of hosts to the infection with microbial pathogens.

The oriental migratory locust *Locusta migratoria manilensis* Meyen is an important pest to many crops worldwide [33]. Neonicotinoids and organophosphate are two important types of insecticides for controlling this pest. However, resistance to such insecticides has become intense in some populations [34,35]. *Metarhizium acridum* and *Beauveria bassiana* have shown potential for the control of several insect pests such as, the cotton bollworm *Helicoverpa zea* and *L. migratoria* [33,36,37]. The mycopesticide “Green Muscle”, which specifically infects the short-horned grasshopper species, has been developed to save crops from locusts. However, the interactions of entomopathogenic fungi with insect hosts have been less frequently evaluated. Thus, we investigated the potential of the new fungal strain *M. anisopliae* CQMa421 for the mortality of *L. migratoria*. We further investigated the immune responses of *L. migratoria* after challenge with *M. anisopliae*. Infection with *M. anisopliae* could cause the immune responses of *L. migratoria*. This study also suggested that infection with the fungus *M. anisopliae* might result in the death of *L. migratoria* adults and nymphs, indicating that *M. anisopliae* might be a potential biocontrol agent to suppress this destructive pest.

## 2. Materials and Methods

### 2.1. M. anisopliae and Insect Culture

The entomopathogenic fungal strain, *M. anisopliae* CQMa421 was isolated from the rice leafroller *Cnaphalocrocis medinalis* and maintained at the China General Microbiological Culture Collection Center (CGMCC, No. 460). The strain used in this study was isolated and cultured in our laboratory (i.e., the Genetic Engineering Research Center, Chongqing University, Chongqing, China). Prior to the experiments, the conidia of *M. anisopliae* were collected after 14 days of growth in 1/4 SDAY medium, which comprises 18 g of agar, 5 g of yeast extract, 10 g of glucose and 2.5 g of peptone per liter of sterilized water. Mycelia were removed by filtration through sterile lens paper. Afterward, the conidia of *M. anisopliae* were diluted into serial concentrations in conjunction with 0.1% Tween 80 and used for subsequent experiments. The concentrations of *M. anisopliae* spores were verified using a Petroff-Hausser counting slide under a microscope. Then, serial conidial concentrations, 1 × 10^5^ conidia/mL, 1 × 10^6^ conidia/mL, 1 × 10^7^ conidia/mL and 1 × 10^8^ conidia/mL, of *M. anisopliae*, were prepared using 0.1% Tween 80.

The individuals of *L. migratoria* used in the study was obtained from the experimental colonies and reared for more than ten years at the Plant Experimental Base of Chongqing University. The locusts were maintained in cages at a temperature 30 ± 3 °C and relative humidity (RH) 50 ± 5% under a 14: 10 h (light: dark) photoperiod. Individual locusts were supplied the fresh maize leaves/ryegrass and wheat bran daily.

### 2.2. The Effect of M. anisopliae CQMa421 on L. migratoria Survival

To examine the potential effects of *M. anisopliae* on *L. migratoria*, the adults and the emerging fifth-instar nymphs of *L. migratoria* were collected for bioassay experiments. Each *L. migratoria* nymph or adult was treated from locust pronotum using 5 μL serial concentrations of *M. anisopliae*. A total of 20 larval or 20 adult individuals as a group were placed into a cage and provided maize leaves, and three replicates were included per treatment group. In the control counterpart, we used 5 μL of 0.1% Tween 80 to treat each individual locust. After treatment, all nymphs and adults were kept in the bioassay room, and their foods were replenished daily. The survival of the locusts was then checked daily and was monitored until the death of all individuals. Individual locusts found dead in the cages were removed and incubated for 10 days to check the conidial formation of locust corpses. We then determined the LT_50_ and the LC_50_ of *M. anisopliae* on locusts after 10 days on the basis of the results of a probit analysis.

### 2.3. Effects on Hemocyte Concentration

To further evaluate the effects of the fungus *M. anisopliae* on the hemocyte concentrations, *L. migratoria* individuals were selected for further examination after infection with the fungus *M. anisopliae* at a concentration 1 × 10^7^ conidia/mL. The hemolymph cells of *L. migratoria* were collected according to the methods carried out by Gillespie et al. [38]. The arthrodial membrane of *L. migratoria* was first swabbed with 70% ethanol and then pierced with a sterile needle. The cells and an equal volume of anticoagulant solution were then immediately mixed together. In this treatment, the hemolymph from ten alive individuals *L. migratoria* was collected (10 μL for each), pooled after 24, 48, 72, and 96 h, and mixed with well-prepared anticoagulant solution. The anticoagulant solution was prepared by consisting of 30 mM sodium citrate, 26 mM citric acid, 100 mM d-glucose, 10 mM EDTA and 60 mM NaCl per 100 mL. The hemocyte concentrations of *L. migratoria* were quantified using a hemocytometer with 10 μL aliquots under a microscope, and five replicates were examined per treatment.

### 2.4. The Enzymatic Activities of ProPO and SOD

The enzymatic activity of ProPO and SOD were tested according to the manufacturer’s instructions after exposure to *M. anisopliae* at a concentration 1 × 10^7^ conidia/mL (Suzhou Comin Biotech Co., Ltd., Suzhou, China). In brief, the hemolymph of 10 *L. migratoria* individuals was collected as described above and was diluted 10 times prior to subsequent experiments. To examine the activities of the ProPO and SOD, the diluted hemolymph was examined using a microplate reader (BioTek Instruments, Inc., Winooski, VT, USA). We evaluated the SOD activity according to the inhibition of the photochemical reduction of nitro blue tetrazolium (NBT) at a wavelength of 560 nm. Each unit of SOD activity was the amount of enzyme that caused a 50% inhibition of the NBT reduction. ProPO can catalyze the catechol to produce the quinones, which absorb light at a wavelength of 525 nm, so the ProPO activity was measured to be 525 nm. The activities of both SOD and ProPO were expressed as units/mg protein.

### 2.5. Immune-Related Gene Expression Induced by M. anispliae

We further evaluated the effects of *M. anisopliae* on the expression of immune-related genes (i.e., defensin, spaetzle and attacin) after fungal infection using quantitative real-time PCR (qRT-PCR). Individual locusts were collected randomly from the *M. anisopliae* treatment or control treatment group and analyzed. Briefly, the total RNA in the locust hemolymph of ten select individuals was extracted by using the Trizol reagent (Invitrogen, Shanghai, China). For this, 1 μg total RNA was reverse-transcribed in a 20-μL reaction using the RT-PCR Kit (TaKaRa, Beijing, China). Then, the iCycler iQ Real-time PCR System (Bio-Rad, Hercules, CA, USA) was selected to perform qRT-PCR with SYBR-Green. The cycling parameters were as follows: 95 °C for 3 min, and 40 cycles of 95 °C for 5 s and 60 °C for 15 s, followed by melting curve generation from 65 to 95 °C. The expression of *β-actin* was selected as to normalize the expression of the immune-related genes according to the 2^−ΔΔ*C*t^ method [39]. All qRT-PCR protocols used the least stringent criteria possible. The primers designed for qRT-PCR in this experiment are listed in Table 1, and three replicates were included per treatment.

### 2.6. Data Analysis

The LT_50_ and LC_50_ of *M. anisopliae* against *L. migratoria* were analyzed using the probit analysis in SPSS 23.0 software. Prior to the analyses, the Shapiro-Wilk test and the Levene test were selected to evaluate the normality and homogeneity of variances, respectively. If the data were not normally distributed, they were normalized or analyzed by the Mann–Whitney U test. Afterward, one-way ANOVA with the least significant difference (LSD) test was applied to examine the effects of *M. anisopliae* on locust LT_50_. The LC_50_ of adult and larva was examined by *t*-test. The hemocyte concentration and the enzymatic activities of SOD and ProPO after the insects were exposed to *L. anisopliae* were analyzed via the *t*-test. The relative expression of the select genes was also analyzed via the *t*-test. The significance level was set at *p* < 0.05.

## 3. Results

### 3.1. Mortality of the Fungus M. anisopliae on L. migratoria

**Figure 1:**
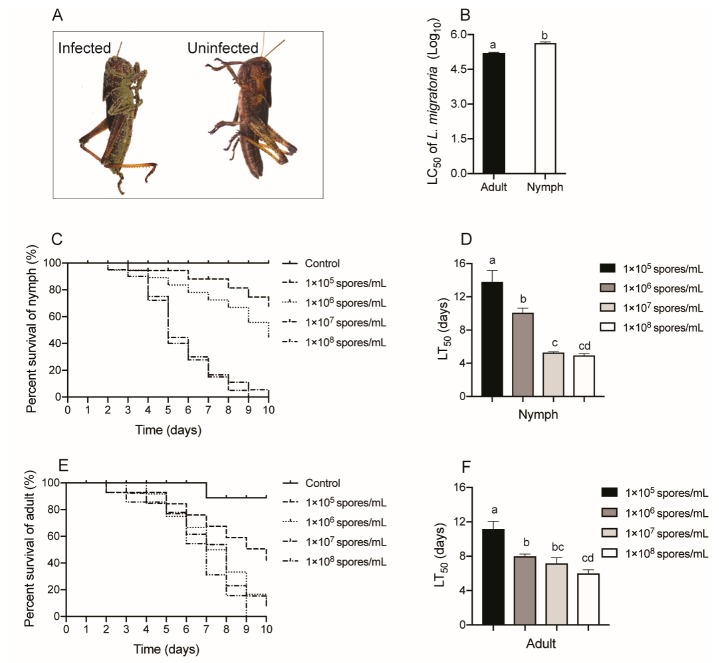
Survival of *L. migratoria* after exposure to *M. anisopliae* CQMa421. (**A**) the corpses of *L. migratoria* insects infected with *M. anisopliae* CQMa421 or not; (**B**) LC_50_ of *L. migratoria* adults and nymphs; (**C**) Survival rate of *L. migratoria* nymphs treated with *M. anisopliae* CQMa421; (**D**) LT_50_ of *L. migratoria* nymphs after being challenged with *M. anisopliae* CQMa421. (**E**) Survival rate of *L. migratoria* adults treated with *M. anisopliae* CQMa421; (**F**) LT_50_ of *L. migratoria* adults after being challenged with *M. anisopliae* CQMa421. The different letters indicate significant differences, and the bars represent the means ± SEs.

The adults and nymphs of *L. migratoria* responded differently to challenge with serial concentrations of the fungus *M. anisopliae* CQMa421. First, we noticed that the corpses of *L. migratoria* insects infected with *M. anisopliae* CQMa421 for 10 days covered the fungal conidia (Figure 1A) but not the controls. The LC_50_ of *L. migratoria* adults and nymphs after infection for 10 days also showed differences (*p* = 0.01, *t*-test; Figure 1B). The LT_50_ of locust nymphs treated with low concentrations of *M. anisopliae*, 1 × 10^5^ conidia/mL or 1 × 10^6^ conidia/mL (13.81 days and 10.09 days, respectively), was higher than that under high concentrations of *M. anisopliae*, 1 × 10^7^ conidia/mL or 1 × 10^8^ conidia/mL (5.30 days and 4.96 days, respectively). The LT_50_ of locust nymphs also showed significant differences after exposure to serial concentrations of *M. anisopliae* (F_3,8_ = 32.735, *p* < 0.001; Figure 1C,D). Similarly, the LT_50_ of *L. migratoria* adults after exposure to concentrations of 1 × 10^5^ conidia/mL was 11.18 days, while the LT_50_ shortened to 6.00 days under 1 × 10^8^ conidia/mL. The LT_50_ of adult locusts showed a significant difference between the serial concentrations of *M. anisopliae* (F_3,8_ = 13.527, *p* = 0.002; Figure 1E,F).

### 3.2. Concentration of Hemocytes

**Figure 2:**
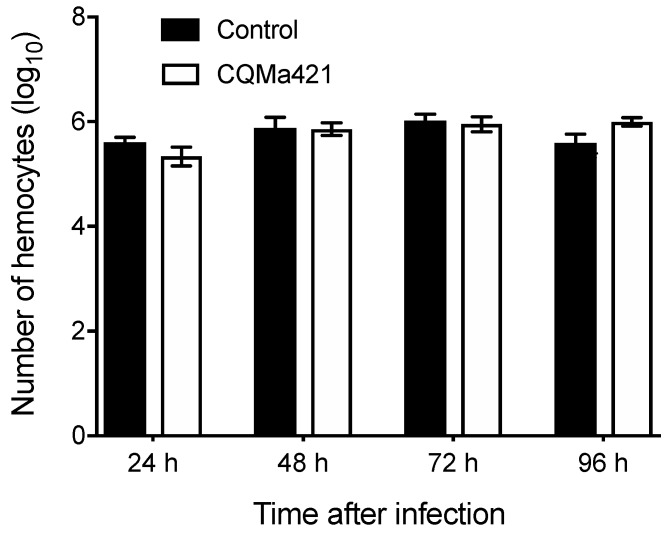
Concentration of hemocytes after challenge with *M. anisopliae* CQMa421 from 24 to 96 h. The bars represent the means ± SEs.

To further investigate the numbers of hemocytes after challenge with *M. anisopliae*, we counted the hemocyte concentration of *L. migratoria* hemolymphs. From 24 to 72 h, the number of hemocytes under the *M. anisopliae* treatment was similar to that under the control treatments (Figure 2). After 96 h of *M. anisopliae* infection, the number of hemocytes under the fungal treatment was greater than that under the controls, there was no statistical difference between the *M. anisopliae* and control treatment, with no significant difference (*p* = 0.061, *t*-test; Figure 2).

### 3.3. Enzymatic Activity

**Figure 3:**
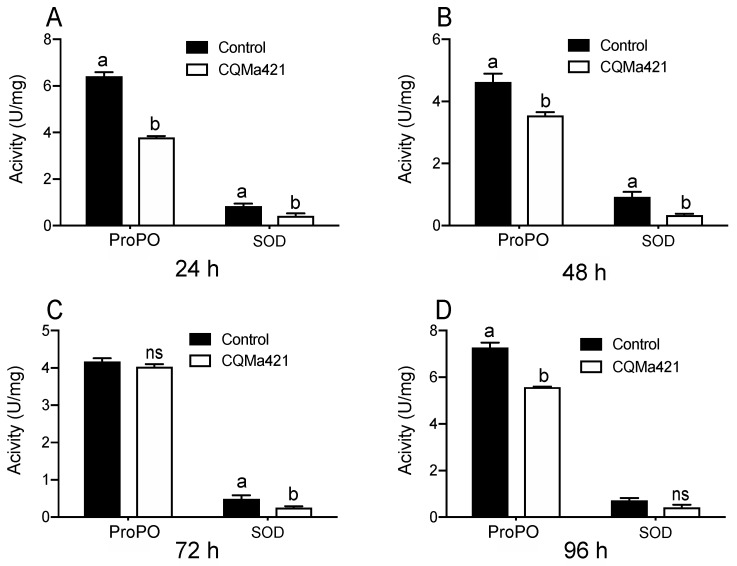
ProPO and SOD activities of *L. migratoria* after challenge with *M. anisopliae* CQMa421. (**A**) Enzymatic activity of *L. migratoria* after treatment with *M. anisopliae* CQMa421 for 24 h; (**B**) Enzymatic activity of *L. migratoria* after treatment with *M. anisopliae* CQMa421 for 48 h; (**C**) Enzymatic activity of *L. migratoria* after treatment with *M. anisopliae* CQMa421 for 72 h; (**D**) Enzymatic activity of *L. migratoria* after treatment with *M. anisopliae* CQMa421 for 96 h. The different letters indicate significant differences, and the bars represent the means ± SEs.

The enzymatic activities of the two enzymes ProPO and SOD varied after the locusts were treated with *M. anisopliae* and were lower than those under the control treatments from 24 h to 48 h (Figure 3A,B). The activities of ProPO at 72 h and SOD at 96 h did not differ from those under the control treatment (*p* = 2.44 and *p* = 0.60 for ProPO and SOD, *t*-test; Figure 3C,D). However, the activities of SOD at 72 h and ProPO at 96 h after exposure to the fungus displayed significant differences compared with those under the control treatment (*p* < 001 and *p* = 0.046 for SOD and ProPO, *t*-test; Figure 3C,D).

### 3.4. Expression of Immune-Related Genes

**Figure 4:**
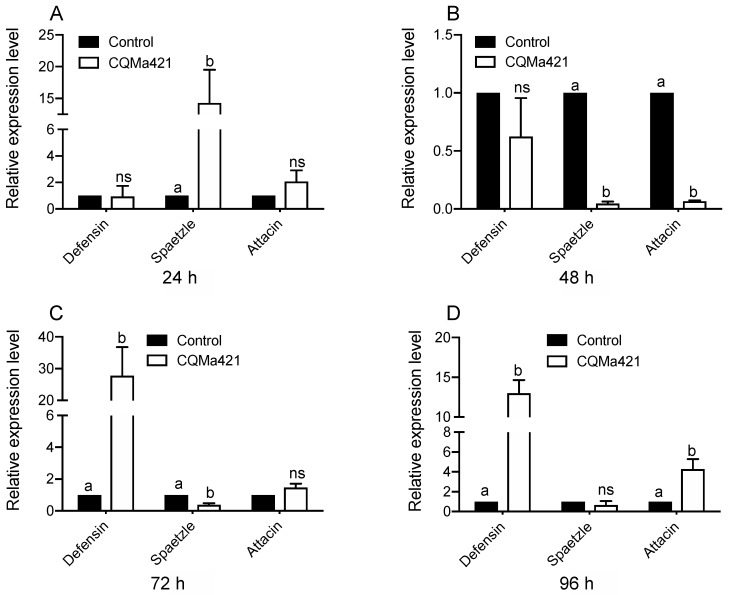
Relative expression of genes after challenge with *M. anisopliae* CQMa421. (**A**) The relative expression of defensin, spaetzle and attacin 24-h post infection; (**B**) the relative expression of defensin, spaetzle, and attacin 48 h post infection; (**C**) the relative expression of defensin, spaetzle and attacin 72 h post infection; (**D**) the relative expression of defensin, spaetzle and attacin 96 h post infection. The different letters indicate significant differences, and the bars represent the means ± SEs.

When *L. migratoria* was infected with the fungus *M. anisopliae*, the gene expression levels showed differences from 24 to 96 h. The expression of the gene spaetzle after exposure to *M. anisopliae* was greater than that under the control treatment at 24 h post infection (*p* < 0.001, *t*-test; Figure 4A), but the defensin and attacin expression did not differ from that under the control treatment. The expression levels of defensin, spaetzle and attacin after 48 h of *M. anisopliae* treatment were low (spaetzle: *p* < 0.001, *t*-test and attacin: *p* < 0.001, *t*-test; Figure 4B). However, after 72 h and 96 h of infection, defensin showed high expression levels, with a similar result observed in attacin at 96 h post infection (Figure 4C,D). In contrast, no significant difference was found in the attacin expression at 72 h and the spaetzli expression at 96 post infection (attacin: *p* = 0.451, *t*-test; spaetzle: 0.132, *t*-test; Figure 4C,D).

## 4. Discussion

*Locusta migratoria* is one of the most persistent agricultural pests [40]. Among the methods used, the chemical pesticides are the most common way to suppress this pest. However, pest populations have become resistant to some insecticides due to their long-term application [41]. Moreover, effects on nontargets (i.e., natural enemies and pollinators) [42], and destructive environmental consequences, and threats to human health have resulted in a desire to decrease the use of such insecticides [43]. Thus, using alternative, environmentally friendly ways to control them and other insect pests is urgent. Fungal insecticides are promising alternatives for the control of insect pests given the lack of fungal-resistance and their environmental safety [44,45]. Several studies have investigated the use of entomopathogenic fungi for the controlling insect pests, including *M. anisopliae* [6,33,46].

Compared to chemical insecticides, entomopathogenic fungi are promising biological control agents for many insect pests and show efficient potential for insecticide-resistant pests with less environmental risk [15,47]. Our results found that *M. anisopliae* CQMa421 could effectively infected the adults and nymphs of the pest *L. migratoria*, suggesting the potential of this fungus for the pest control. *Aspergillus oryzae* (Eurotiales: Trichocomaceae) was also reported as an entomopathogenic fungus for the control of the locust *L. migratoira* [48]. The low LT50 of *L. migratoria* found under concentrations of 1 × 10^7^ conidia/ml and 1 × 10^8^ conidia/mL indicated high susceptibility of *L. migratoria* to *M. anisopliae* infection. Several other studies also reported the similar results, with an increased susceptibility under high conidial concentrations [49,50]. However, some of the infected locust individuals in our study were not dead after 10 days of treatment. The surviving locust individuals may be partially tolerant to the fungus *M. anisopliae*. In addition, the susceptibility of insect pests to entomopathogenic fungi may vary under different environmental conditions [51].

The hemocytes of insect hosts play important roles in mediating the production of cellular defenses and soluble effector molecules (i.e., encapsulation and phagocytosis) [52]. These organisms can produce different types of hemocytes when they face different invasions depending on the substances present [53,54]. Several studies have shown that hosts produce different numbers or types of hemocytes after infection with fungal strains of *M. anisopliae* and *M. acridum* [53,55]. Hosts also respond differently to different strengths of fungal infection [53]. The different responses of hosts to different external stimuli indicate that hosts have different response functions to such challenges [56]. Entomopathogenic fungi infect insect pests directly via the host cuticle [44], while the chemical thiamethoxam has different routes, including by physical contact, stomach action or systemic poison [57]. In addition, entomopathogenic fungi affect gut bacterial genera, which is one of the major factors leading to host death [58]. However, it is unknown whether the chemicals cause the death of the host due to changes in bacterial genera. In this study, we challenged *L. migratoria* with *M. anisopliae* to evaluate the immune responses of hosts. However, we noticed that the number of hemocytes in the *M. anisopliae* treatment was similar to that in the controls. In a previous study, the number of hemocytes also had no significant changes after 72 h of infection with the fungus *M. acridum* in *L. migratoria*, but a reduced number was observed after 96 h and 120 h [53].

The level of enzymatic activity in the host reflects the physiological activity of the host. Different stimuli may elicit responses from different enzymes and different levels of activity. Hosts may respond differently to the challenges with different substances, and even to the same enzymes [59]. Host protease and chitinase enzymes are usually initially expressed at high levels after exposure to entomopathogenic fungi [44]. During this process, the entomopathogenic fungi can penetrate the cuticle of the host via assistance from such related enzymes. However, the immune system of hosts is activated in response to fungal penetration, and the amount of phagocytes increases during this period [53]. In contrast, detoxifying enzymes become activated when hosts are challenged with chemical insecticides [6]. The immune responses of the cabbage looper *Trichoplusiani* host to baculovirus challenge suggest a dose- and time-dependent infection [60]. The integration of an insecticidal scorpion toxin (Bjα IT) gene into *Metarhizium acridum* can improve the fungal virulence to *L. migratoria* by growing quickly in the locust hemolymph, which may reduce the immune responses of the locust [61]. The results of this study showed that the enzymatic activities of ProPO and SOD differed after the insects were challenged with the fungus *M. anisopliae* for 24 h. The level of enzymatic activity tended to decrease in the fungal treatment, indicating that the ProPO of host *L. migratoria* might be inhibited during this period.

The expression levels of the genes defensin, spaetzle and attacin differed after challenge with *M. anisopliae*. Organismal immunity and antioxidants play important roles in defense against harmful chemicals, pathogenic microorganisms and parasites [55,62]. Studies of *L. migratoria* have identified 470 immune-related genes, 58 of which were differentially expressed in hemocytes and fat bodies after infection with the fungus *Metarhizium acridum* [63]. However, hosts may respond differently to different challenges. Our results showed that *L. migratoria* also presented different gene expression levels in response to challenge with fungus *M. anisopliae*, with high expression occurring from 24 h, 72 h and 48 h (for spaetzle). However, the expression level under the treatment varied with time, indicating that the host displayed different immune responses in terms of duration of exposure.

## 5. Conclusions

The fungus *M. anisopliae* could effectively infect *L. migratoria* and affect the immune responses of locust hosts. The study provides insights into the interactions of insect hosts and entomopathogenic fungi and suggests that the fungus *M. anisopliae* CQMa421 is a potential prospect for controlling this pest.

## Figures and Tables

**Table 1:** PCR primers used in this study.

Primers	Primer Sequences (5’-3’)
*Spaetzle*-F	AGCTTGTGGGTACGGAGAC
*Spaetzle*-R	GGGCGATGAATAGATGAAAC
*Defensin*-F	GCGTCTGTCTCCTCTG
*Defensin*-R	CCCTTGTAGCCCTTGTT
*Attacin*-F	GTGCTCCTCGTCGTTCTGA
*Attacin*-R	CCCACGCCTTTCTCTCTGT
*β-actin*-F	GCAGCCAGCAACCAGGAG
*β-actin*-R	ACCATCTGTCCACGGATAATAGC

F, forward primer; R, reverse primer.

## References

[1] Shah P.A., Pell J.K. (2003). Entomopathogenic fungi as biological control agents. Appl. Microbiol. Biotechnol..

[2] Vega F.E., Goettel M.S., Blackwell M., Chandler D., Jackson M.A., Keller S., Koike M., Maniania N.K., Monzón A., Ownley B.H. (2009). Fungal entomopathogens: New insights on their ecology. Fungal Ecol..

[3] De Faria M.R., Wraight S.P. (2007). Mycoinsecticides and Mycoacaricides: A comprehensive list with worldwide coverage and international classification of formulation types. Biol. Control.

[4] Mascarin G.M., Lopes R.B., Delalibera I., Fernandes E.K.K., Luz C., Faria M. (2019). Current status and perspectives of fungal entomopathogens used for microbial control of arthropod pests in Brazil. J. Invertebr. Pathol..

[5] Zimmermann G. (2007). Review on safety of the entomopathogenic fungus Metarhizium Anisopliae. Biocontrol Sci. Technol..

[6] Jia M., Cao G., Li Y., Tu X., Wang G., Nong X., Whitman D.W., Zhang Z. (2016). Biochemical basis of synergism between pathogenic fungus Metarhizium anisopliae and insecticide chlorantraniliprole in Locusta migratoria (Meyen). Sci. Rep..

[7] Zimmermann G. (2010). The entomopathogenic fungus Metarhizium anisopliae and its potential as a biocontrol agent. Pestic. Sci..

[8] Tang J., Liu X., Ding Y., Jiang W., Xie J. (2019). Evaluation of Metarhizium anisopliae for rice planthopper control and its synergy with selected insecticides. Crop Prot..

[9] Lee M.R., Li D., Lee S.J., Kim J.C., Kim S., Park S.E., Baek S., Shin T.Y., Lee D.H., Kim J.S. (2019). Use of Metarhizum aniopliae s.l. to control soil-dwelling longhorned tick, Haemaphysalis longicornis. J. Invertebr. Pathol..

[10] Larsen A.E., Gaines S.D., Deschênes O. (2017). Agricultural pesticide use and adverse birth outcomes in the San Joaquin Valley of California. Nat. Commun..

[11] Verger P.J.P., Boobis A.R. (2013). Reevaluate pesticides for food security and safety. Science.

[12] Gerage J.M., Meira A.P.G., da Silva M.V. (2017). Food and nutrition security: Pesticide residues in food. Nutrire.

[13] Kohler H.R., Triebskorn R. (2013). Wildlife ecotoxicology of pesticides: Can we track effects to the population level and beyond?. Science.

[14] Whalon M.E., Mota-Sanchez D., Hollingworth R.M. (2008). Global Pesticide Resistance in Arthropods.

[15] Knols B.G., Bukhari T., Farenhorst M. (2010). Entomopathogenic fungi as the next-generation control agents against malaria mosquitoes. Future Microbiol..

[16] Xu J., Xu X., Shakeel M., Li S., Wang S., Zhou X., Yu J., Xu X., Yu X., Jin F. (2017). The Entomopathogenic fungi Isaria fumosorosea plays a vital role in suppressing the immune system of Plutella xylostella: RNA-Seq and DGE analysis of immunity-related genes. Front. Microbiol..

[17] Söderhäll K., Cerenius L. (1998). Role of the prophenoloxidase-activating system in invertebrate immunity. Curr. Opin. Immunol..

[18] Arya B., Pier Adelchi R., Leifer C.A., Elena K., Alexander S., Oleg C., Shirakawa A.K., Farber J.M., Segal D.M., Oppenheim J.J. (2002). Toll-like receptor 4-dependent activation of dendritic cells by beta-defensin 2. Science.

[19] Mullen L.M., Goldsworthy G.J. (2006). Immune responses of locusts to challenge with the pathogenic fungus Metarhizium or high doses of laminarin. J. Insect Physiol..

[20] Wang C., Leger R.J.S. (2006). A collagenous protective coat enables Metarhizium anisopliae to evade insect immune responses. Proc. Natl. Acad. Sci. USA.

[21] Ali S., Zhen H., Ren S. (2010). Production of cuticle degrading enzymes by Isaria fumosorosea and their evaluation as a biocontrol agent against diamondback moth. J. Pest Sci..

[22] Cerenius L., Thörnqvist P.O., Vey A., Johansson M.W., Söderhäll K. (1990). The effect of the fungal toxin destruxin E on isolated crayfish haemocytes. J. Insect Physiol..

[23] Gopalakrishnan S., Chen F.Y., Thilagam H., Qiao K., Xu W.F., Wang K.J. (2011). Modulation and interaction of immune-associated parameters with antioxidant in the immunocytes of Crab scylla paramamosain challenged with lipopolysaccharides. Evid. Based Complement. Altern. Med..

[24] Müller P., Donnelly M.J., Ranson H. (2007). Transcription profiling of a recently colonised pyrethroid resistant *Anopheles gambiae* strain from Ghana. BMC Genom..

[25] Tsukamoto T., Ichimaru Y., Kanegae N., Watanabe K., Yamaura I., Katsura Y., Funatsu M. (1992). Identification and isolation of endogenous insect phenoloxidase inhibitors. Biochem. Biophys. Res. Commun..

[26] Cerenius L., Söderhäll K. (2004). The prophenoloxidase-activating system in invertebrates. Immunol. Rev..

[27] Isabelle V.G., Bruno L., Frédéric B. (2008). Bacterial strategies to overcome insect defences. Nat. Rev. Microbiol..

[28] Araújo J.P.M., Hughes D.P. (2016). Chapter one—Diversity of entomopathogenic fungi: Which groups conquered the insect body?. Adv. Genet..

[29] Lu H.-L., Leger R.S. (2016). Insect immunity to entomopathogenic fungi. Adv. Genet..

[30] Kounatidis I., Ligoxygakis P. (2012). Drosophila as a model system to unravel the layers of innate immunity to infection. Open Biol..

[31] Cociancich S., Ghazi A., Hetru C., Hoffmann J.A., Letellier L. (1993). Insect defensin, an inducible antibacterial peptide, forms voltage-dependent channels in Micrococcus luteus. J. Biol. Chem..

[32] Hultmark D., Engström A., Andersson K., Steiner H., Bennich H., Boman H.G. (1983). Insect immunity. Attacins, a family of antibacterial proteins from Hyalophora cecropia. EMBO J..

[33] Peng G., Wang Z., Yin Y., Zeng D., Xia Y. (2008). Field trials of Metarhizium anisopliae var. acridum (Ascomycota: Hypocreales) against oriental migratory locusts, Locusta migratoria manilensis (Meyen) in Northern China. Crop Prot..

[34] Yang M.L., Zhang J.Z., Zhu K.Y., Xuan T., Liu X.J., Guo Y.P., Ma E.B. (2010). Mechanisms of organophosphate resistance in a field population of oriental migratory locust, Locusta migratoria manilensis (Meyen). Arch. Insect Biochem. Physiol..

[35] Yang H. (2002). Preliminary study on the resistance of *Locusta migratoria manilensis* to malathion. Plant Prot. Technol. Ext..

[36] Delgado F.X., Britton J.H., Lobolima M.L. (2012). Field and laboratory evaluations of leading entomopathogenic fungi isolated from Locusta migratoria capito sauss in madagascar. Mem. Entomol. Soc. Can..

[37] Lopez D.C., Sword G.A. (2015). The endophytic fungal entomopathogens Beauveria bassiana and Purpureocillium lilacinum enhance the growth of cultivated cotton (Gossypium hirsutum) and negatively affect survival of the cotton bollworm (Helicoverpa zea). Biol. Control.

[38] Gillespie J.P., Burnett C., Charnley A.K. (2000). The immune response of the desert locust Schistocerca gregaria during mycosis of the entomopathogenic fungus, Metarhizium anisopliae var acridum. J. Insect Physiol..

[39] Pfaffl M.W. (2001). A new mathematical model for relative quantification in real-time RT-PCR. Nucleic Acids Res..

[40] Xie J., Li S., Zhang W., Xia Y. (2019). RNAi-knockdown of the Locusta migratoria nuclear export factor protein results in insect mortality and alterations in gut microbiome. Pest Manag. Sci..

[41] Ahmad M. (2008). Potentiation between pyrethroid and organophosphate insecticides in resistant field populations of cotton bollworm Helicoverpa armigera (Lepidoptera: Noctuidae) in Pakistan. Pestic. Biochem. Physiol..

[42] Whitehorn P.R., Goulson D. (2012). Neonicotinoid pesticide reduces bumble bee colony growth and queen production. Science.

[43] Jin S.F., Feng M.G., Chen J.Q. (2008). Selection of global Metarhizium isolates for the control of the rice pest Nilaparvata lugens (Homoptera: Delphacidae). Pest Manag. Sci..

[44] St Leger R., Screen S. (2001). Fungi as Biocontrol Agents: Progress, Problems and Potential.

[45] Jorgensen P.S., Aktipis A., Brown Z., Carriere Y., Downes S., Dunn R.R., Epstein G., Frisvold G.B., Hawthorne D., Grohn Y.T. (2018). Antibiotic and pesticide susceptibility and the Anthropocene operating space. Nat. Sustain..

[46] Wang C., Wang S. (2017). Insect pathogenic fungi: Genomics, molecular interactions, and genetic improvements. Annu. Rev. Entomol..

[47] Bahiense T.C., Fernandes E.K., Angelo Ida C., Perinotto W.M., Bittencourt V.R. (2008). Performance of Metarhizium anisopliae and Its combination with deltamethrin against a pyrethroid-resistant strain of Boophilus microplus in a stall test. Anim. Biodivers. Emerg. Dis. Predict. Prev..

[48] Zhang P.F., You Y.W., Song Y., Wang Y.Z., Zhang L. (2015). First record of Aspergillus oryzae (Eurotiales: Trichocomaceae) as an entomopathogenic fungus of the locust, Locusta migratoria (Orthoptera: Acrididae). Biocontrol Sci. Technol..

[49] Kirkland B.H., Cho E.M., Keyhani N.O. (2004). Differential susceptibility of Amblyomma maculatum and Amblyomma americanum (Acari: Ixodidea) to the entomopathogenic fungi Beauveria bassiana and Metarhizium anisopliae. Biol. Control.

[50] Shrestha G., Enkegaard A., Steenberg T. (2015). Laboratory and semi-field evaluation of Beauveria bassiana (Ascomycota: Hypocreales) against the lettuce aphid, Nasonovia ribisnigri (Hemiptera: Aphididae). Biol. Control.

[51] Wraight S.P., Ugine T.A., Ramos M.E., Sanderson J.P. (2016). Efficacy of spray applications of entomopathogenic fungi against western flower thrips infesting greenhouse impatiens under variable moisture conditions. Biol. Control.

[52] Strand M.R. (2010). The insect cellular immune response. Insect Sci..

[53] Yu Y., Cao Y., Xia Y., Liu F. (2016). Wright-Giemsa staining to observe phagocytes in Locusta migratoria infected with Metarhizium acridum. J. Invertebr. Pathol..

[54] Duressa T.F., Vanlaer R., Huybrechts R. (2015). Locust cellular defense against infections: Sites of pathogen clearance and hemocyte proliferation. Dev. Comp. Immunol..

[55] Cao G., Jia M., Zhao X., Wang L., Tu X., Wang G., Nong X., Zhang Z. (2016). Different effects of Metarhizium anisopliae Strains IMI330189 and IBC200614 on enzymes activities and hemocytes of *Locusta migratoria* L.. PLoS ONE.

[56] Smith R.C., Carolina B.M., Marcelo J.L. (2015). Hemocyte differentiation mediates the mosquito late-phase immune response against Plasmodium in Anopheles gambiae. Proc. Natl. Acad. Sci. USA.

[57] Castle S.J., Byrne F.J., Bi J.L., Toscano N.C. (2010). Spatial and temporal distribution of imidacloprid and thiamethoxam in citrus and impact on Homalodisca coagulata populations. Pest Manag. Sci..

[58] Wei G., Lai Y., Wang G., Chen H., Li F., Wang S. (2017). Insect pathogenic fungus interacts with the gut microbiota to accelerate mosquito mortality. Proc. Natl. Acad. Sci. USA.

[59] Li Z., Li M., He J., Zhao X., Chaimanee V., Huang W.F., Nie H., Zhao Y., Su S. (2017). Differential physiological effects of neonicotinoid insecticides on honey bees: A comparison between Apis mellifera and Apis cerana. Pestic. Biochem. Physiol..

[60] Scholefield J.A., Shikano I., Lowenberger C.A., Cory J.S. (2019). The impact of baculovirus challenge on immunity: The effect of dose and time after infection. J. Invertebr. Pathol..

[61] Peng G.X., Xia Y.X. (2015). Integration of an insecticidal scorpion toxin (Bj alpha IT) gene into Metarhizium acridum enhances fungal virulence towards Locusta migratoria manilensis. Pest Manag. Sci..

[62] Dubovskiy I.M., Kryukov V.Y., Yaroslavtseva O.N., Levchenko M.V., Belgibaeva A.B., Adilkhankyzy A., Glupov V.V. (2012). The activity of nonspecific esterases and glutathione-S-transferase in Locusta migratoria larvae infected with the fungus Metarhizium anisopliae (Ascomycota, Hypocreales). Entomol. Rev..

[63] Zhang W., Chen J., Keyhani N.O., Zhang Z., Li S., Xia Y. (2015). Comparative transcriptomic analysis of immune responses of the migratory locust, Locusta migratoria, to challenge by the fungal insect pathogen, Metarhizium acridum. BMC Genom..

